# Effective Resolution of Postoperative Stoma Edema With Repeated Application of Topical 50% Glucose: A Case Report

**DOI:** 10.7759/cureus.90800

**Published:** 2025-08-23

**Authors:** Satoshi Ueno, Tsuyoshi Suzuki, Mitsuko Suzuki, Shigeru Marubashi, Ken Iseki

**Affiliations:** 1 Department of Emergency and Critical Care Medicine, Fukushima Medical University, Fukushima, JPN; 2 Department of Hepatobiliary Pancreatic and Transplant Surgery, Fukushima Medical University, Fukushima, JPN

**Keywords:** advanced pressure ulcer, glucose solution, hip disarticulation, stoma edema, type 2 diabetes mellitus

## Abstract

Although glucose-induced osmotic gradients have been used to reduce intestinal edema, no standardized technique has been established. Repeated applications of this method have not been documented, and concerns remain regarding its potential effects on glycemic control. We present a case of a patient in his 20s admitted for treatment of an advanced pressure ulcer over the left greater trochanter. His medical history included spina bifida and type 2 diabetes mellitus. The ulcer was complicated by osteonecrosis of the left femur, and a left hip disarticulation was planned as a life-saving procedure.

Before this procedure, a sigmoid colostomy was performed to divert the fecal stream. On postoperative day six following colostomy, the hip disarticulation was performed, and the patient's general condition gradually improved. However, persistent stoma edema was observed 12 days after surgery. Congestive discoloration of the stoma mucosa developed, and complications such as stomal outlet obstruction and abdominal distension were suspected. To address this complication, 20 mL of 50% glucose solution was applied topically to the intestinal mucosa eight times over five days. Following this treatment, a marked reduction in stomatal edema was observed. Notably, the procedure used in our case did not adversely affect perioperative glycemic control, suggesting its feasibility as a minimally invasive approach for managing acute-phase stoma edema.

## Introduction

Postoperative intestinal edema following stoma creation is one of the early complications of surgery. It is generally expected to improve within approximately one week [1]. However, in critically ill patients in intensive care settings - particularly those with coexisting inflammatory diseases - persistent edema may occur due to ongoing postoperative inflammation [1]. The edema can hinder stoma self-care [2] and delay the detection of complications such as mucocutaneous separation and peristomal dermatitis [3]. When this condition is prolonged, it may lead to mucosal injury [4] and increase the risk of further complications, including stoma prolapse and stomal outlet obstruction. Therefore, appropriate management aimed at reducing intestinal edema is desirable. However, in the intensive care setting, such interventions are often limited by challenges in maintaining fluid balance.

We report a case where persistent postoperative stoma edema was successfully managed through multiple topical applications of a 50% glucose solution directly on the intestinal mucosa. Written informed consent for publication was obtained from the patient.

## Case presentation

A 28-year-old male patient presented with a medical history of spina bifida, lower limb paralysis, and type 2 diabetes mellitus. He initially presented to a local clinic with a pressure ulcer in the left greater trochanter. Although outpatient treatment was initiated, the ulcer gradually worsened and was accompanied by fever, elevated inflammatory markers, and poor oral intake. The patient was admitted to a local hospital for further management.

Owing to the progression of the ulcer and suspected gas gangrene and osteomyelitis of the left femoral trochanteric region, he was transferred to our hospital. Ulcer debridement was performed on admission; however, the femoral head and trochanter exhibited extensive sequestration. Considering the severity of the infection, limb preservation was deemed infeasible, and left hip disarticulation was planned as a life-saving procedure.

Given the ulcer’s proximity to the anus and the anticipated difficulty with postoperative self-care due to lower limb paralysis, there was a concern regarding fecal contamination of the surgical wound. Therefore, a double-barrel sigmoid colostomy was performed before hip disarticulation to divert the fecal stream.

On postoperative day six after colostomy, the left hip disarticulation was performed, and wound care was continued. Oral intake was resumed on postoperative day two. However, the patient complained of nausea and abdominal distention after hip disarticulation. Abdominal X-ray revealed bowel dilatation extending from the transverse colon to the stoma outlet. Although mild stoma mucosa edema was observed immediately after colostomy, the edema did not improve and gradually worsened with discoloration, suggesting venous congestion.

**Figure 1:**
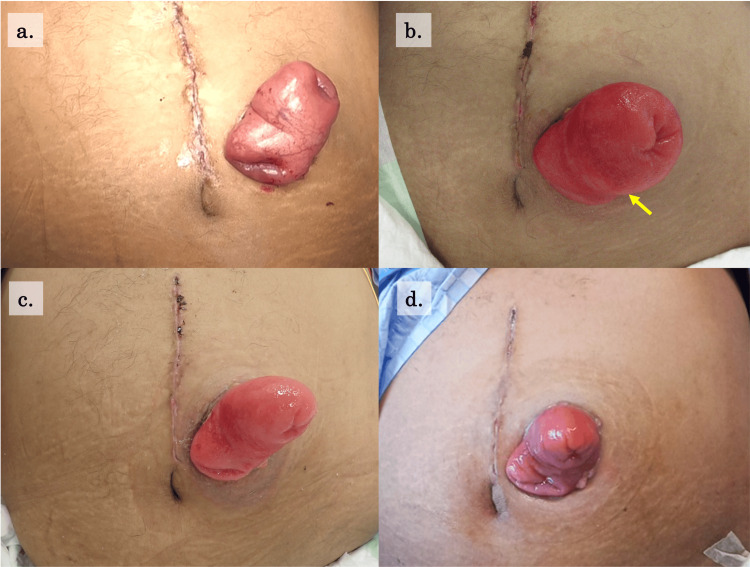
Changes in stoma appearance a. Postoperative ay zero b. Postoperative day 10 (before the procedure for stoma); L×W: 50mm×48mm, changes in mucocutaneous coloration can be seen c. Postoperative day 16 (after the procedure for stoma); L×W: 47mm×43mm d. Postoperative day 22

**Figure 2:**
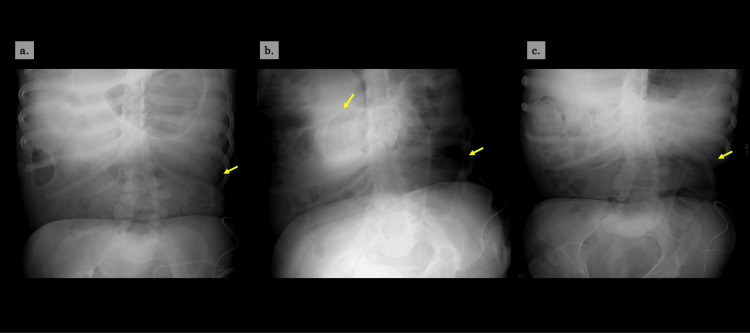
Changes in abdominal X-ray findings a. Postoperative day 7, no bowel dilatation b. Postoperative day 11 (before initiation of the stoma procedure), bowel dilatation extending c. Postoperative day 13 (after initiation of the stoma procedure), improvement trend in bowel dilatation

By postoperative day 12, the edema had not resolved, raising concern about potential stomal outlet obstruction due to persistent swelling. In response, we initiated treatment with a topical 50% glucose solution to reduce the edema. On day 12, 20 mL of 50% glucose solution (two ampoules) was applied directly to the stoma mucosa. This intervention was followed by twice-daily applications of 20 mL (one ampoule) each morning and evening. The glucose solution was administered through the stoma pouch outlet using a 20 mL syringe, allowing several minutes of contact before draining into a waste collection bag. Over five days, up to postoperative day 16, a total of eight applications were performed. Compared with the pretreatment condition, a noticeable improvement in stoma edema was observed (Figure 1). In addition, flatus was observed after the treatment, and abdominal X-ray showed no worsening of bowel dilatation (Figure 2). Of note, although the stoma edema had improved, a CT scan obtained on postoperative day 24 showed no evidence of muscular layer thickening or other findings that could have caused stomal obstruction.

**Figure 3:**
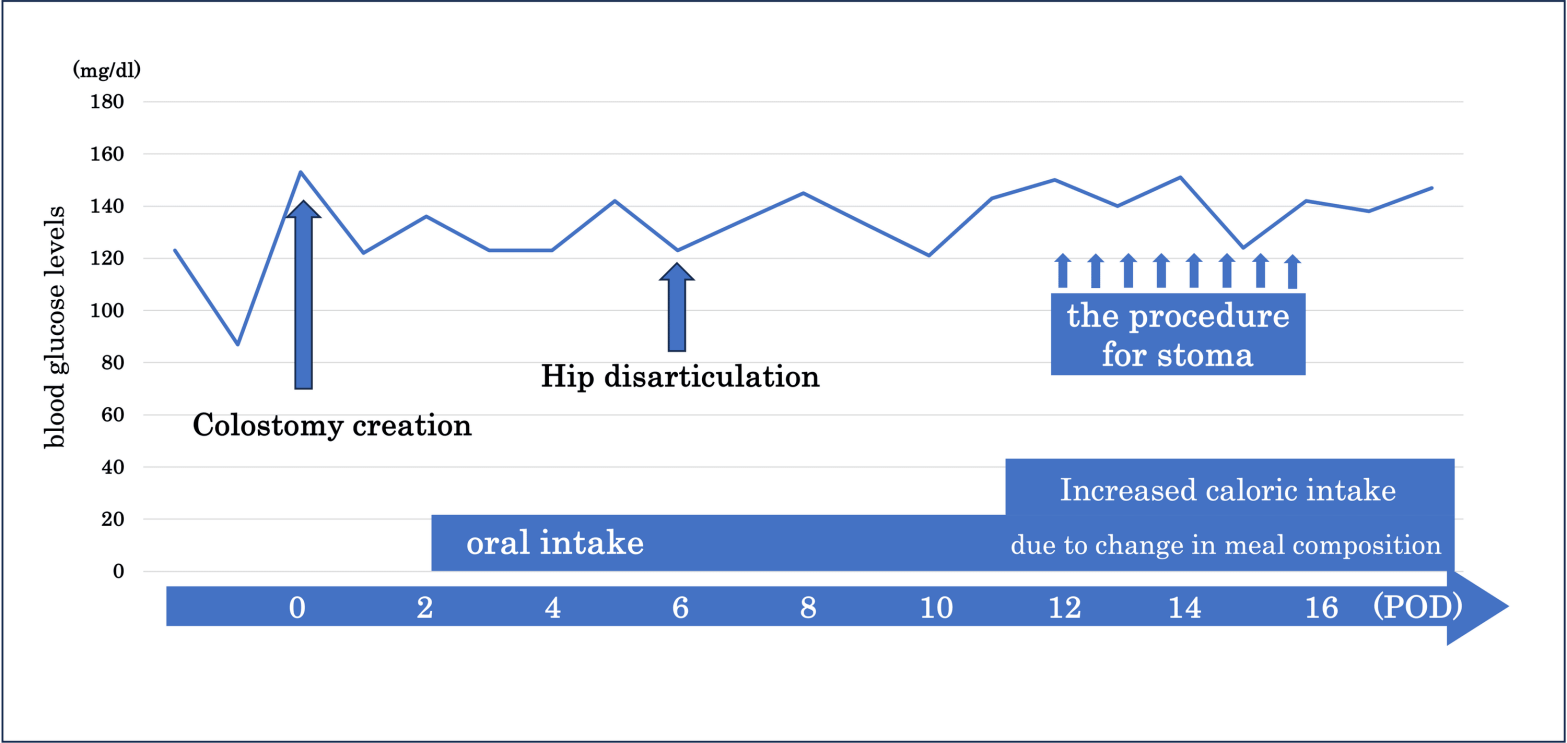
Changes in early morning blood glucose levels Oral intake was resumed on postoperative day two after colostomy. Hip disarticulation was performed on postoperative day six. Caloric intake increased from 1400 kcal to 1950 kcal on postoperative day 11. The stoma procedure was initiated on the evening of postoperative day 12 and continued until the morning of postoperative day 16. Eight procedures were performed on the morning of postoperative day 16.

Considering the patient’s diagnosis of type 2 diabetes mellitus, blood glucose levels were continuously monitored during the perioperative period. Although nutritional intake varied slightly around the time of surgery, no deterioration in glycemic control was observed during the five-day treatment period (Figure 3).

The stoma edema continued to improve, and the patient was subsequently transferred to another department for continued postoperative wound care following the hip disarticulation.

## Discussion

In cases where postoperative stoma edema emerges during the acute phase and contributes to complications such as mucocutaneous separation or stomal outlet obstruction, topical application of glucose solution to the stoma mucosa may be an effective treatment option. Postoperative stoma edema is generally attributed to impaired venous perfusion of the intestine caused by surgical trauma [5]. In the absence of arterial ischemia, this edema type typically resolves spontaneously within three to seven days after surgery [1,3], making active intervention unnecessary in most cases. However, in our case, the edema persisted beyond postoperative day 12 and was accompanied by progressive mucosal discoloration, suggesting worsening venous congestion. Moreover, stoma edema can hinder early postoperative self-care [2] and delay the detection and management of complications such as mucocutaneous separation and peristomal dermatitis [3]. In our case, edema obscured signs of worsening mucocutaneous separation, prompting the decision to initiate active intervention. Additionally, the patient’s abdominal distension and nausea raised concerns about possible stomal outlet obstruction, further justifying the use of localized edema reduction to relieve the symptoms. In patients receiving intensive care such as in this case, persistent stoma edema can lead to complications like mucocutaneous separation or stomal outlet obstruction, which are occasionally difficult to manage because they complicate treatment. Thus, this minimally invasive technique, with its potential for symptomatic relief, was considered a promising option.

Furthermore, in cases where stoma edema is caused by a complex condition requiring intensive care, repeated glucose applications may be a more effective intervention. While this technique has limited efficacy in habitual stoma prolapse, previous reports have demonstrated its benefits in a single application for cases of sudden stoma prolapse or stomas that are difficult to revert [6]. In our case, the technique was applied to transient postoperative stoma edema, which likely contributed to its effectiveness, with repeated applications further enhancing edema reduction. The prolonged stoma edema was likely due to surgical trauma from stoma creation, severe inflammation from a long-standing pressure ulcer infection, and additional surgical stress from hip disarticulation. Given these multiple contributing factors, the stoma edema was considered to have resulted from a complex etiology. Repeated applications of the technique produced a more stable and sustained reduction in localized swelling compared with a single intervention. While the natural improvement in the patient’s general condition may have contributed to the edema resolution, several observations suggest a significant effect of this technique. The edema persisted longer than expected, worsened despite signs of systemic improvement prior to treatment, and showed rapid improvement following the intervention. These findings suggest that the technique played a substantial role in relieving stoma-associated venous congestion. Regarding the appropriate duration of this technique, further accumulation of cases will be required to establish clear recommendations. However, in patients receiving intensive care, where maintaining fluid balance is often difficult, it may be reasonable to continue the procedure until macroscopic improvement of the edema is observed or stoma-related problems caused by the edema are resolved.

This technique promotes excretion of excess water from the intestinal mucosa through osmotic gradients. However, no standardized consensus currently exists regarding the type of solution used or the frequency of application. Thus, further accumulation of cases and clinical evaluations is necessary. Previous reports have described the use of a 50% glucose solution for the treatment of stoma prolapse [6], an intervention also adopted in our case. The osmotic pressure of a 50% glucose solution is significantly higher than that of normal saline, with an osmotic ratio ranging from approximately 9.8 to 10.7, facilitating the removal of excess water from the stoma via its high osmolarity [6]. Furthermore, a 50% glucose solution is widely available, making this procedure easily applicable in many healthcare settings. From a multidisciplinary care perspective, this technique is advantageous regarding standardization and implementation within clinical teams. Various agents, including powdered sugar, 50% glucose solution, and 10% glucose solution, have been reported for similar purposes [2,7-10]; however, further detailed evaluations are needed to determine the efficacy and safety of each option. Notably, in Japan, the intraluminal application of glucose solution to the intestine constitutes off-label use. In our case, the off-label status of the procedure was fully explained to the patient, and informed consent was obtained before its implementation. Although off-label, the technique is minimally invasive, not systemically administered, and technically simple when applied externally, enhancing its clinical feasibility.

Previous reports have raised concerns regarding potential fluctuations in blood glucose levels due to glucose absorption [2], particularly in patients with diabetes, for whom careful consideration is needed, given its potential impact on glycemic control. However, in our case, despite multiple applications of the technique, no significant changes were observed in the fasting morning blood glucose levels before and after the intervention. While the colon primarily reabsorbs water and has minimal glucose absorption capacity, posing a low risk of hyperglycemia from topical glucose application, the small intestine, which actively absorbs glucose, may present a higher risk. Thus, in cases involving small-bowel stomas, the potential impact on glycemic trends must be evaluated with greater caution. To date, previous reports have not extensively discussed blood glucose fluctuations associated with this technique. Therefore, further case accumulation and careful glycemic monitoring are essential for broader clinical applications.

## Conclusions

Topical application of a 50% glucose solution to the intestinal mucosa effectively reduced postoperative intestinal edema following stoma creation. This technique may be particularly beneficial in cases where postoperative edema persists, such as in patients requiring intensive care. Importantly, no deterioration in glycemic control was observed before or after the procedure. The minimally invasive nature of the technique and its low risk of complications are also considered notable advantages.
